# Introduction of Modified BglBrick System in *Lactococcus lactis* for Straightforward Assembly of Multiple Gene Cassettes

**DOI:** 10.3389/fbioe.2021.797521

**Published:** 2021-12-10

**Authors:** Tina Vida Plavec, Tim Ključevšek, Aleš Berlec

**Affiliations:** ^1^ Department of Biotechnology, Jožef Stefan Institute, Ljubljana, Slovenia; ^2^ Faculty of Pharmacy, University of Ljubljana, Ljubljana, Slovenia

**Keywords:** *Lactococcus lactis*, genetic engineering, BglBrick, multifunctional bacteria, expression system

## Abstract

Genetic modification of lactic acid bacteria is an evolving and highly relevant field of research that allows the engineered bacteria to be equipped with the desired functions through the controlled expression of the recombinant protein. Novel genetic engineering techniques offer the advantage of being faster, easier and more efficient in incorporating modifications to the original bacterial strain. Here, we have developed a modified BglBrick system, originally introduced in *Escherichia coli* and optimized it for the lactic acid bacterium *Lactococcus lactis*. Six different expression cassettes, encoding model proteins, were assembled in different order as parts of a modified BglBrick system in a novel plasmid pNBBX. All cassettes included nisin promoter, protein encoding gene and transcription terminator. We demonstrated successful intracellular expression of the two fluorescent proteins and display of the four protein binders on the bacterial surface. These were expressed either alone or concomitantly, in combinations of three model proteins. Thus, a modified BglBrick system developed herein enables simple and modular construction of multigene plasmids and controlled simultaneous expression of three proteins in *L. lactis*.

## Introduction

Lactic acid bacteria (LAB) are considered safe and valuable host organisms in biotechnology with various potential applications. Their use as hosts for the expression of heterologous proteins that act as biosensors, biocatalysts and even therapeutics has been demonstrated (Stahl and Uhlen, 1997; Lee et al., 2003; Nguyen et al., 2016; Plavec and Berlec, 2019). Various engineering tools are available for efficient protein expression, secretion and/or their surface display through different anchoring domains (Michon et al., 2016; Plavec et al., 2019).

The introduction of biotechnological modifications into LAB can be achieved by plasmid-encoded expression systems (Plavec and Berlec, 2019). Plasmids offer the advantage of providing multiple gene copies in a simple way and the possibility of using inducible promoters to achieve better control over protein expression. The most commonly used system for inducible expression in *L. lactis* is the nisin-controlled gene expression system (NICE), for which several plasmids have been developed and made commercially available. The best known are pNZ8048 and its variant pNZ8148 (Mierau and Kleerebezem, 2005). Recently, pNZDual with two nisin promoters for simultaneous inducible expression of two recombinant proteins has been developed (Berlec et al., 2018).

The novel genetic engineering systems for *L. lactis* are usually introduced by following the example of such systems in Gram-negative bacterium *Escherichia coli*, for which diverse cloning and expression tools are available. Some have already been introduced in *L. lactis*. The high-throughput ligation-independent cloning was achieved in *L. lactis* by the use of a vector backbone exchange (Geertsma and Poolman, 2007). The TA-cloning system based on the PCR amplification of the NICE-plasmid pNZ8148 was optimized for *L. lactis* (Berlec and Strukelj, 2012). Gateway cloning technology for *L. lactis* is also based on NICE. It enables transfer of DNA fragments from a donor vector to any gateway-compatible vectors by highly specific recombination reactions, thereby avoiding the use of restriction enzymes (Douillard et al., 2011). For engineering more complex gene networks, the Gibson assembly method has been applied in LAB (Kong et al., 2016). However, there are some drawbacks associated with the newly developed systems. TA-cloning can lead to misguided inserts (Niarchos et al., 2017), number of backbone vectors for gateway platform is limited in addition to limited possibility for combination with other recombination systems (Reece-Hoyes and Walhout, 2018), while Gibson assembly loses efficiency as the number of inserts increases (Roth et al., 2014). Therefore, to overcome the limitations of current methods, novel straightforward genetic engineering systems are needed.

The BglBrick genetic engineering system (Anderson et al., 2010) originates from the BioBrick standard (Knight, 2003), which was the first to describe the assembly of standard biological parts in a single reaction. In comparison to BioBrick, BglBrick is a more flexible and reliable system and allows efficient assembly of large composite parts in *E. coli*. Iterative restrictions and ligations rely on the use of the restriction sites BglI and BamHI with compatible overhangs. The result of the reaction is a 6-nucleotide scar sequence (GGATCT) that encodes glycine-serine (Anderson et al., 2010). BglBrick has been successfully used for the construction of protein expression systems, formation of multidomain fusion proteins and targeted gene integration into the *E. coli* genome (Anderson et al., 2010). A collection of 96 BglBrick-compatible plasmids with a combination of replication origins, antibiotic resistance genes, and inducible promoters was created to simplify engineering and provide better control (Lee et al., 2011). BglBrick system also allowed for easier formation of nanobody multimers (Goldman et al., 2017) and has been adapted for use in *Burkholderia sacchari* (Guaman et al., 2018).

In this study, we developed a modified BglBrick system for the assembly of multiple gene cassettes in *L. lactis*. Due to the presence of BamHI restriction site in most of our plasmids used for the assembly, we adapted the original BglBrick system by replacing BamHI with BclI restriction site that also creates compatible overhang. Expression cassettes with different functional elements coding for six model proteins were used to test the modified BglBrick system. We demonstrated the assembly of different combinations of expression cassettes, successful expression of all individual model proteins, and controlled simultaneous expression of three proteins in *L. lactis*.

## Materials and Methods

### Bacterial Strains, Media and Growth Conditions

The bacterial strains used in this study are listed in Table 1. *L. lactis* NZ9000 was grown in M-17 medium (Merck, Kenilworth, NJ, United States) containing 0.5% glucose (GM-17) and chloramphenicol (10 µg/mL) at 30°C without aeration. Biliverdin HCl (15.5 µg/mL; Sigma-Aldrich, St. Louis, MO, United States) was added for infrared fluorescent protein (IRFP) expression.

**TABLE 1:** Primers and plasmids used in this study.

Strain, plasmid, primer or gene	Relevant features or sequence	References
Primers		
BX-R-TT	AAA​AAA​CTC​GAG​ATA​TTG​ATC​AAA​CGA​TTA​TGC​CGA​TAA​CTA​AAC	This work
NB-F-PnisA	AAA​AAA​GCT​AGC​ATA​TAG​ATC​TAG​TCT​TAT​AAC​TAT​ACT​GAC	This work
Xho-F-8148	TTA​AAA​CTC​GAG​AAA​ACA​GGC​GTT​ATC​GTA​G	This work
Nhe-R-8148	TAA​TAA​GCT​AGC​CTG​TAA​TAT​AAA​AAC​CTT​CTT​CAA​C	This work
Plasmids		
pNZ8148	pSH71 derivative, P_*nisA*_ *,* Cm^r^, nisin-controlled expression	Kuipers et al. (1993); de Ruyter et al. (1996); Mierau and Kleerebezem (2005)
pSD-fHER2	pNZ8148 containing gene fusion of *spUsp45*, *flagtag*, *z-her2* and *acmA3b*	Plavec et al. (2021)
pSD-AffEpCAM	pNZ8148 containing gene fusion of *spUsp45*, *aff-epcam* and *acmA3b*	Plavec et al. (2021)
pSD-mycEva	pNZ8148 containing gene fusion of *sp*_Usp45_, *myctag*, *eva3* and *acmA3b*	Zahirović et al., manuscript in preparation
pSD-ZIL	pNZ8148 containing gene fusion of *sp*_Usp45_, *ZIL6* and *acmA3b*	Zahirović et al., manuscript in preparation
pNZ-IRFP	pNZ8148 containing *irfp713*	Berlec et al. (2015)
pNZ-mCh	pNZ8048 containing *mCherry*	Dr. Jorge G. Gomez-Gutierrez, (Martinez-Jaramillo et al., 2017)
pNBBX	pNZ8148 containing NheI, BglII, BclI and XhoI restriction sites	This work
pNBBX-fHER2	pNBBX containing HER cassette	This work
pNBBX-AffEpCAM	pNBBX containing EpC cassette	This work
pNBBX-mycEva	pNBBX containing Eva cassette	This work
pNBBX-ZIL	pNBBX containing ZIL cassette	This work
pNBBX-IRFP	pNBBX containing IRFP cassette	This work
pNBBX-mCh	pNBBX containing mCh cassette	This work
p-fHER2-mycEva	pNBBX containing HER and Eva cassettes	This work
p-mycEva-fHER2	pNBBX containing Eva and HER cassettes	This work
p-fHER2-ZIL	pNBBX containing HER and ZIL cassettes	This work
p-ZIL-fHER2	pNBBX containing ZIL and HER cassettes	This work
p-EpCAM-mycEva	pNBBX containing EpC and Eva cassettes	This work
p-mycEva-EpCAM	pNBBX containing Eva and EpC cassettes	This work
p-EpCAM-ZIL	pNBBX containing EpC and ZIL cassettes	This work
p-ZIL-EpCAM	pNBBX containing ZIL and EpC cassettes	This work
p-fHER2-mycEva-IRFP	pNBBX containing HER, Eva and IRFP cassettes	This work
p-mycEva-fHER2-IRFP	pNBBX containing Eva, HER and IRFP cassettes	This work
p-fHER2-ZIL-IRFP	pNBBX containing HER, ZIL and IRFP cassettes	This work
p-ZIL-fHER2-IRFP	pNBBX containing ZIL, HER and IRFP cassettes	This work
p-EpCAM-mycEva-IRFP	pNBBX containing EpC, Eva and IRFP cassettes	This work
p-mycEva-EpCAM-IRFP	pNBBX containing Eva, EpC and IRFP cassettes	This work
p-EpCAM-ZIL-IRFP	pNBBX containing EpC, ZIL and IRFP cassettes	This work
p-ZIL-EpCAM-IRFP	pNBBX containing ZIL, EpC and IRFP cassettes	This work
p-fHER2-mycEva-mCh	pNBBX containing HER, Eva and mCh cassettes	This work
p-mycEva-fHER2-mCh	pNBBX containing Eva, HER and mCh cassettes	This work
p-fHER2-ZIL-mCh	pNBBX containing HER, ZIL and mCh cassettes	This work
p-ZIL-fHER2-mCh	pNBBX containing ZIL, HER and mCh cassettes	This work
p-EpCAM-mycEva-mCh	pNBBX containing EpC, Eva and mCh cassettes	This work
p-mycEva-EpCAM-mCh	pNBBX containing Eva, EpC and mCh cassettes	This work

### Molecular Cloning

Plasmid DNA was isolated using NucleoSpin Plasmid (Macherey and Nagel, Düren, Germany), with an additional lysozyme treatment step for *L. lactis*. Lactococci were transformed by electroporation using a Gene Pulser II device (Biorad, Hercules, CA, United States) according to MoBiTec GmbH (Goettingen, Germany) instructions. Nucleotide sequencing was performed by Microsynth AG (Balgach, Switzerland).

**FIGURE 1:**
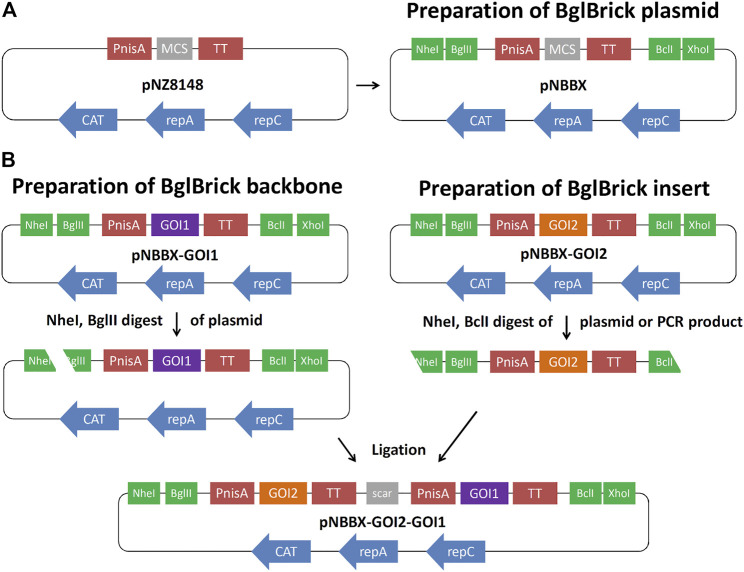
Preparation of BglBrick plasmid pNBBX by insertion of NheI/BglII and BclI/XhoI sites into pNZ8148 **(A)**. Example of upstream BglBrick cloning of a gene cassette using NheI/BglII for the backbone and NheI/BclI for the insert and exploiting BglII/BclI complementarity resulting in a scar **(B)**. GOI, gene of interest; PnisA, nisin promoter; TT, transcription terminator; MCS, multiple cloning site.

Plasmid pNZ8148 was modified by inserting restriction enzyme recognition sites NheI and BglII upstream of the nisin promoter (PnisA), and restriction enzyme recognition sites BclI and XhoI downstream of the transcription terminator (TT), resulting in plasmid pNBBX (Figure 1A). This was achieved by amplifying the pNZ8148 region between PnisA and TT by PCR with KOD Hot Start Polymerase (Sigma-Aldrich) using the primers NB-F-PnisA and BX-R-TT, and the pNZ8148 backbone using the primers Xho-F-8148 and Nhe-R-8148 (Table 1). The two amplicons were ligated *via* newly introduced NheI and XhoI sites at their termini. The amplicon from plasmid pNZ8148 was first cloned into the pGEM-T Easy plasmid. Gene cassettes for BglBrick constructs, containing PnisA, genes of interest and TT were amplified from pSD-fHER2 (cassette fHER2), pSD-AffEpCAM (cassette AffEpCAM), pSD-mycEva (cassette mycEva), pSD-ZIL (cassette ZIL6), pNZ-IRFP (cassette IRFP) and pNZ-mCh (cassette mCherry), by PCR with KOD Hot Start Polymerase using the insert-independent universal primers NB-F-PnisA and BX-R-TT (Table 1). These amplicons were subcloned to plasmid pNBBX *via* the NheI/XhoI restriction sites to obtain pNBBX-fHER2, pNBBX-AffEpCAM, pNBBX-mycEva, pNBBX-ZIL, pNBBX-IRFP and pNBBX-mCh. These plasmids were then used to assemble multiple cassette plasmids, p-fHER2-mycEva-IRFP, p-mycEva-fHER2-IRFP, p-fHER2-ZIL-IRFP, p-ZIL-fHER2-IRFP, p-EpCAM-mycEva-IRFP, p-mycEva-EpCAM-IRFP, p-EpCAM-ZIL-IRFP, p-ZIL-EpCAM-IRFP, p-fHER2-mycEva-mCh, p-mycEva-fHER2-mCh, p-fHER2-ZIL-mCh, p-ZIL-fHER2-mCh, p-EpCAM-mycEva-mCh, p-mycEva-EpCAM-mCh, by using different combinations of these amplicons either upstream (restriction with NheI/BglII on the backbone and NheI/BclI on the insert; Figure 1B) or downstream (restriction with BclI/XhoI on the backbone and BglII/XhoI on the insert) of the first insert.

### Expression of Fusion Proteins in *L. lactis*

Overnight cultures of *L. lactis* containing the appropriate plasmids were diluted (1:100) in 10 mL of fresh GM-17 medium and grown to optical density A_600_ = 0.8–1.0. Fusion protein expression was induced with 25 ng/mL nisin (Fluka AG, Buchs, Switzerland) for 3 h at 30°C. After incubation, the culture was kept at 4°C for flow cytometric analysis and fluorescence measurement.

### Fluorescence Measurement

Aliquots of bacterial cell cultures (200 µL) containing plasmids with IRFP or mCherry were transferred into black, flat-bottomed 96-well plates (Greiner, Kremsmünster, Austria). Fluorescence was measured using a microplate reader (Infinite M1000; Tecan, Männedorf, Switzerland), with excitation and emission at 690 and 713 nm for IRFP-containing constructs (Berlec et al., 2015) and with excitation and emission at 587 and 610 nm for mCherry containing constructs, respectively. The measurements were performed in two technical replicates.

### Flow Cytometry

Flow cytometry was used to assess surface display of protein binders fHER2, mycEva, ZIL6 and AffEpCAM. Bacterial culture in the stationary phase (20 μL) was added to 500 μL of Tris-buffered saline (TBS; 50 mM Tris-HCl, 150 mM NaCl, pH 7.5; or 200 μL PBS for AffEpCAM) and centrifuged at 5,000 x *g* and 4°C for 5 min.

The *L. lactis* pellet was resuspended in 250 μL TBS containing FLAG-tag rabbit polyclonal antibody (Proteintech Group, Chicago, IL, United States; 1:500), MYC-tag mouse polyclonal antibody (Proteintech Group, Chicago, IL, United States; 1:500), 200 μL of TBS containing biotin-conjugated recombinant human IL-6 (ImmunoTools, Friesoythe, Germany; 1:200) or 200 μL PBS with 1 μg/mL recombinant human EpCAM/TROP-1 Fc chimera (R&D systems, Minneapolis, MN, United States), for the detection of fHER2, mycEva, ZIL6 and AffEpCAM, respectively. After 2 h of incubation at room temperature with constant shaking at 100 rpm, cells were washed three times with 200 μL TBS containing 0.1% Tween-20 (0.1% TBST) and resuspended in 250 μL TBS with anti-rabbit IgG Fab2 Alexa Fluor 488 (Cell signaling technology, Danvers, MA, United States; 1:2,000), anti-mouse IgG Fab2 Alexa Fluor 488 (Cell Signaling technology, Danvers, MA, United States; 1:2,000), mouse anti-biotin antibody (Abcam, Cambridge, United Kingdom; 1:1,000) or Alexa Fluor 488 anti-human Fcγ specific antibody (1:500; Jackson ImmunoResearch) for fHER2, mycEva, ZIL6 and AffEpCAM, respectively. For ZIL6, mouse anti-biotin antibody was subsequently detected with anti-mouse IgG Fab2 Alexa Fluor 488 (Cell Signaling technology; 1:1,000). After 2 h of incubation at room temperature with constant shaking at 100 rpm, cells were washed three times with 200 μL 0.1% TBST and finally resuspended in 500 μL TBS. For AffEpCAM, PBS and PBST were used instead of TBS and TBST throughout the procedure.

Samples were analyzed with a flow cytometer (FACS Calibur; Becton Dickinson, Franklin Lakes, United States) using excitation at 488 nm and emission at 530 nm in the FL1 channel. The geometric mean fluorescence intensity (MFI) of at least 20,000 bacterial cells in the corresponding gate was measured. MFI corresponded to the extent of surface display (and expression) of protein binders. The average of at least three independent experiments was considered. FlowJo software was used for data analysis.

### Statistical Analyses

Statistical analyses were performed using GraphPad Prism 6 software. Data are presented as mean ± standard deviation. Student’s *t*-tests were used to detect significant differences between the model protein-expressing *L. lactis* and the empty plasmid control.

## Results

### BglBrick Plasmid Construction

To facilitate cloning in *L. lactis*, while maintaining nisin-controlled expression, we constructed the pNBBX plasmid (Figure 1A) by inserting four additional restriction sites into pNZ8148 (Mierau and Kleerebezem, 2005). By using PCR, NheI and BglII restriction sites were inserted at the 5′ end of the segment containing nisin promoter, multiple cloning site and transcription terminator, while BclI and XhoI restriction sites were inserted at its 3′ end. Likewise, the BglBrick plasmid backbone was amplified from pNZ8148, thereby inserting XhoI at its 5′ end and NheI at its 3′ end. pNBBX was obtained by ligating the BglBrick segment and the BglBrick plasmid backbone.

**FIGURE 2:**
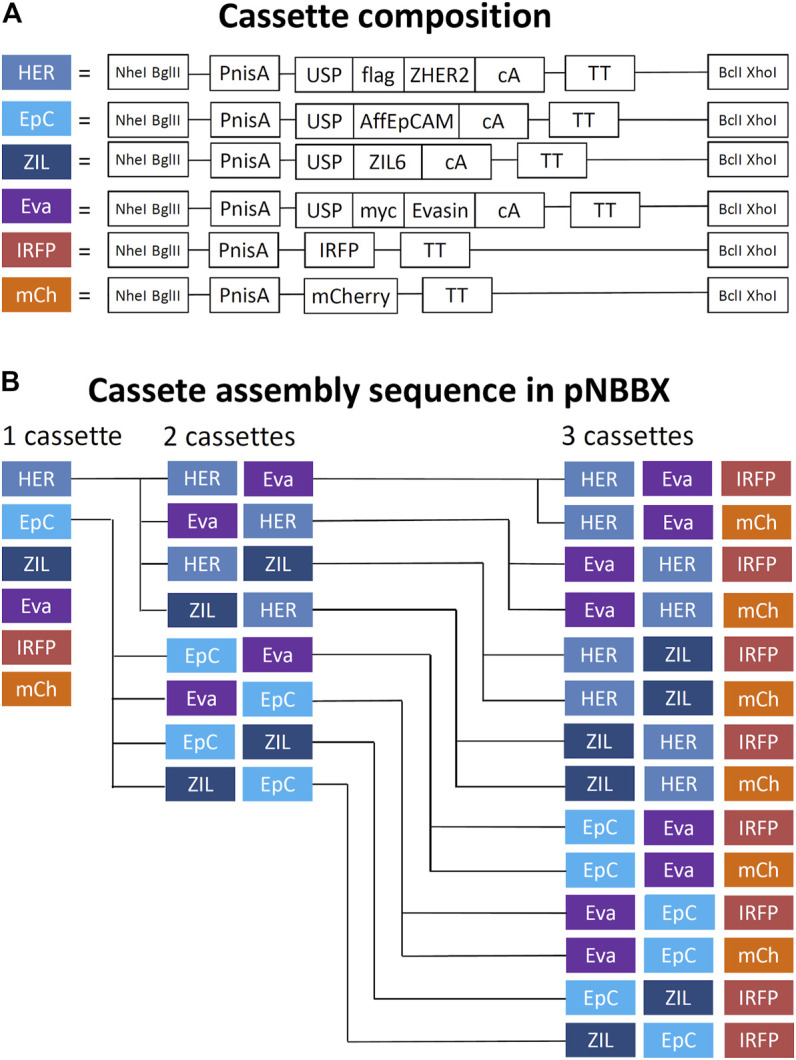
Schematic representation of components of gene cassettes for model recombinant protein expression and surface display **(A)** and an evolutionary tree of successive assembly of multiple gene cassettes in pNBBX plasmids depicted in 5′ to 3′ direction **(B)**. PnisA, nisin promoter; USP, secretion signal; AffEpCAM, EpCAM-targeting affitin; ZHER2, HER2-targeting affibody; Evasin, CXCL-8-binding evasin 3; ZIL6, IL-6-binding affibody; IRFP, infrared fluorescent protein; mCherry, red fluorescent protein; cA, cAcmA anchoring domain; flag, FLAG-tag; myc, MYC-tag; TT, transcription terminator.

By using universal primers BX-R-TT and NB-F-PnisA, gene cassettes containing nisin promoter, gene of interest and transcription terminator (up to 1.460 bp in length) were amplified from pNZ8148 or pNZ8048 derivatives and inserted into pNBBX. For construction of the multiple gene cassette pNBBX, plasmids gene cassettes were inserted upstream of the first cassette using the restriction enzyme pairs NheI/BglI and NheI/BclI, or downstream using the restriction enzyme pairs XhoI/BglI and XhoI/BclI. Altogether, 28 plasmids were constructed using the BglBrick assembly and verified by sequencing (Table 1). Both upstream and downstream assembly was applied, and the evolutionary tree depicting the construction of plasmids containing multiple gene cassettes is shown in Figure 2. Model proteins included four protein binders (affibody against HER2 (Orlova et al., 2006; Feldwisch et al., 2010), affitin against EpCAM (Kalichuk et al., 2018), evasin-3 against IL-8 (Škrlec et al., 2017) and affibody against IL-6 (Zahirović et al., manuscript in preparation) and two fluorescent proteins (IRFP (Berlec et al., 2015) and mCherry (Martinez-Jaramillo et al., 2017)).

### Concomitant Expression and Surface Display of Protein Binders and IRFP

**FIGURE 3:**
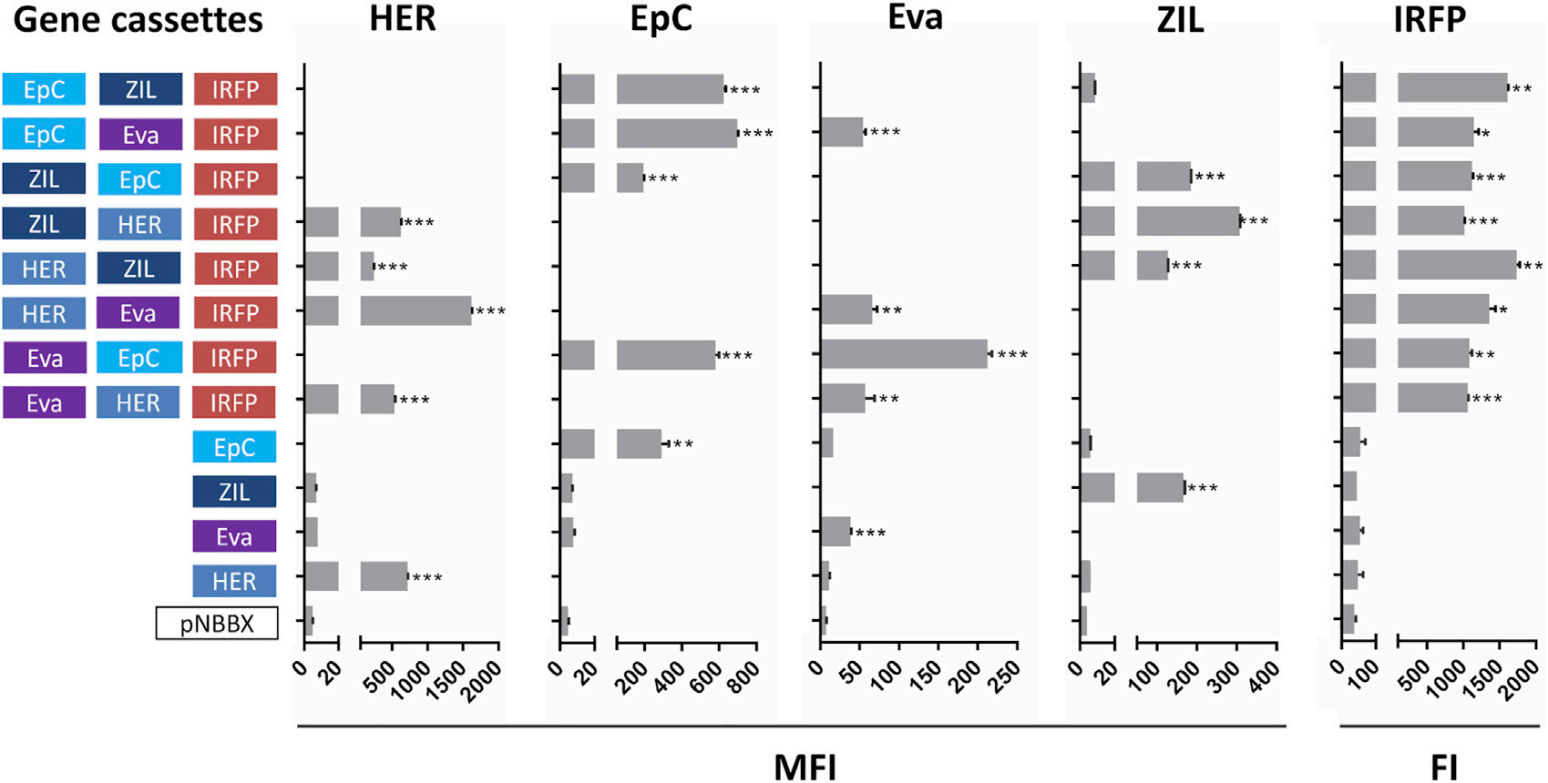
Surface display of individual protein binders (cassettes HER, EpC, Eva and ZIL) assessed by flow cytometry (mean fluorescence intensity, MFI), and expression of fluorescent protein IRFP (cassette IRFP) assessed by measuring fluorescence (fluorescence intensity, FI) in *L. lactis* containing pNBBX with up to three gene cassettes (denoted in 5′-3′ direction). *p < 0.01, **p < 0.001, ***p < 0.0001.

Expression of protein binders from plasmids containing one and two gene cassettes was confirmed by flow cytometry (Supplementary Figure S1). However, more focus was made on our evaluation of protein expression from plasmids containing three gene cassettes. This is a novelty and holds considerable application potential in *L. lactis* engineering. Expression from plasmids containing three gene cassettes was compared to that from plasmids containing one gene cassette, while empty plasmid pNBBX was used as a negative control. In the first set of experiments, surface display of protein binders in the BglBrick plasmids was evaluated by flow cytometry, and expression of IRFP was evaluated by measuring fluorescence. Expression of all model proteins was confirmed (Figure 3). Dot plots of the flow cytometry data and the gating strategy used to generate Supplementary Figure S1 and Figure 3 are presented in Supplementary Figures S2, S3, respectively.

Significant expression and display of the protein binder ZHER2 on the surface of *L. lactis* was confirmed *via* FLAG tag with all plasmids containing HER cassettes, while no expression was detected in control plasmids. Expression and surface display of HER on *L. lactis* was up to 4-fold higher from plasmid p-fHER2-mycEva-IRFP in comparison to other plasmids containing HER cassette. The surface display of the protein binder AffEpCAM was assessed by its ability to bind to the recombinant human receptor EpCAM fused to the Fc region of human IgG. All variants of *L. lactis* containing EpC cassette bound recombinant human EpCAM significantly. Somewhat surprisingly, higher surface display was observed with plasmids containing EpC cassette and two other gene cassettes than with plasmids containing only EpC cassette. Expression and surface display of the protein binder Evasin was confirmed *via* MYC tag in all *L. lactis* variants transformed with plasmids containing Eva cassette. *L. lactis* containing p-mycEva-EpCAM-IRFP exhibited up to 5-fold higher surface display than other variants containing Eva cassette. Low level of nonspecific antibody binding was observed in control *L. lactis* strains*.* The surface display of protein binder ZIL6 was tested by its ability to bind to human recombinant IL-6 conjugated to biotin. The expression of ZIL6 was confirmed in all variants of *L. lactis* containing ZIL cassette except for *L. lactis* containing p-EpCAM-ZIL-IRFP in which the display was practically undetectable. Expression of IRFP was confirmed by fluorescence intensity measurements in all recombinant *L. lactis* that contained the IRFP cassette. No expression was observed with the control plasmid pNBBX and controls without IRFP cassette (Figure 3).

### Concomitant Expression and Surface Display of Protein Binders and mCherry

**FIGURE 4:**
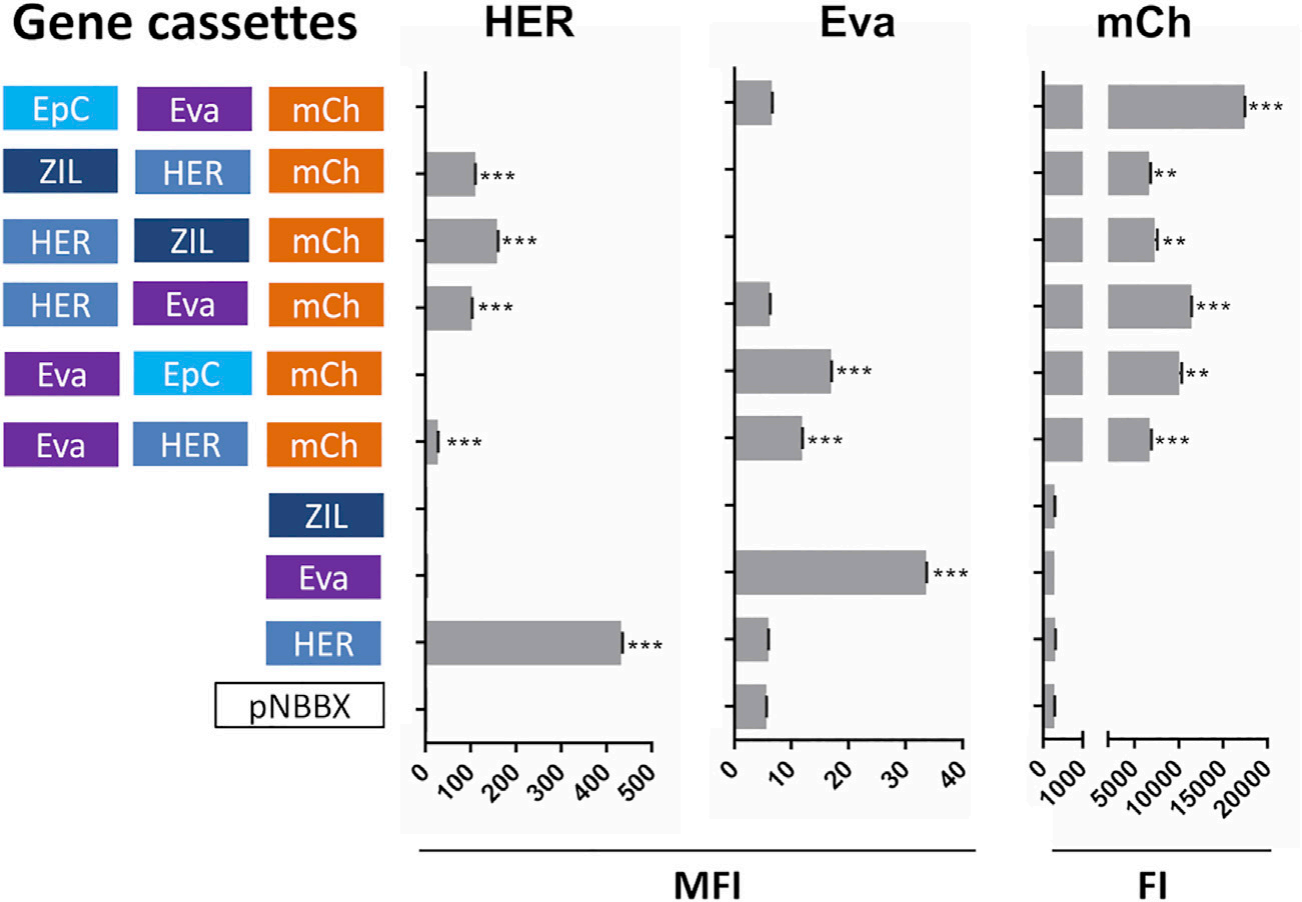
Surface display of selected individual protein binders (cassettes HER and Eva), evaluated by flow cytometry (mean fluorescence intensity, MFI), and expression of fluorescent protein mCherry (cassette mCh); assessed by measuring fluorescence (fluorescence intensity, FI) in *L. lactis* containing pNBBX derivatives with up to three gene cassettes (denoted in 5′-3′ direction). **p < 0.001, ***p < 0.0001.

In the second set of experiments, constructed plasmids containing mCh cassette were evaluated. Apart from monitoring the expression of mCherry, the evaluation focused on the expression of protein binders ZHER2 and Evasin that were present in all genetic constructs and that enabled more straightforward detection with specific antibodies. Similar to plasmids containing IRFP, mCherry, ZHER2 and Evasin were expressed from the plasmids containing their corresponding cassettes (Figure 4). Dot plots of the flow cytometry data and the gating strategy used to generate Figure 4 are presented in Supplementary Figure S4.

## Discussion

Lactic acid bacteria have been used as hosts for heterologous protein expression in various biotechnological and therapeutic applications. For the model LAB *Lactococcus lactis* several methods have been developed to achieve rapid and efficient recombinant protein expression, secretion or surface display (Plavec and Berlec, 2019). Plasmid-based genetic engineering systems in *L. lactis* are mostly based on the use of restriction endonucleases.

In this study, we introduced a modified BglBrick system for straightforward and modular assembly of multiple genes in *L. lactis* to facilitate its genetic engineering. Concomitant expression of multiple proteins can endow *L. lactis* with multiple functions at the same time, which would be beneficial in both industrial applications and therapy. BglBrick was originally developed for *E. coli* (Anderson et al., 2010), and has since been used for various applications (Lee et al., 2011; Goldman et al., 2017; Guaman et al., 2018); however, to our knowledge, its implementation in LAB *L. lactis* has not been reported yet. Our goal was to demonstrate the flexibility of the BglBrick system by assembling different combinations of at least three gene cassettes in different order in *L. lactis* that will allow concomitant expression of at least three proteins. This represents an advantage over the previously reported plasmid pNZDual (Berlec et al., 2018) that allows the simultaneous expression of two proteins in *L. lactis*.

Six gene expression cassettes composed of promoter, gene of interest, and transcription terminator were selected for the assembly and were used to test the functionality of modified BglBrick system. Model proteins were either protein binders that were expressed and subsequently displayed on the surface of *L. lactis* [affitin against EpCAM, affibody against HER2 (Plavec et al., 2021), affibody against IL-6 (Zahirović et al., manuscript in preparation), evasin against IL-8 (Škrlec et al., 2017)] or fluorescent proteins that were expressed intracellularly [infrared fluorescent protein (IRFP) (Berlec et al., 2015), red fluorescent protein (mCherry) (Martinez-Jaramillo et al., 2017)].

We successfully constructed 8 pNBBX plasmids that contained various combinations of two gene cassettes and 14 pNBBX plasmids that contained combinations of three gene cassettes, whereby both upstream and downstream cloning was used. Correct plasmid assembly was confirmed by nucleotide sequencing. The concomitant expression of all proteins was confirmed by flow cytometry or fluorescence measurement. The expression of individual proteins differed between different genetic constructs and depended on the genetic neighborhood. For most of the assemblies, the protein surface display was highest when the gene was positioned in the first position of the genetic construct, which is in line with previous observations (Berlec et al., 2018).

To broaden the general applicability of the gene assemblies, additional variants containing red fluorescent protein mCherry were prepared in combination with selected protein binders. The surface display of selected protein binders from plasmids containing mCherry cassette was confirmed; however, the absolute MFI measured was considerably lower than that observed from plasmids containing IRFP cassette. This could be due to the flow cytometry method that favored relative rather than absolute comparison between different experiments; an observation confirmed with additional assessment of plasmids containing two gene cassettes (Supplementary Figure S1).

In the present study, we achieved the straightforward assembly of multiple gene cassettes in a single pNBBX plasmid by using the modified BglBrick system and confirmed successful expression of multiple proteins. Constructed pNBBX plasmid thus represents a valuable tool for faster genetic engineering and effective protein expression in *L. lactis*.

## Data Availability

The raw data supporting the conclusions of this article will be made available by the authors, without undue reservation.
