# Supplementary material for: Introduction of Modified BglBrick System in Lactococcus lactis for Straightforward Assembly of Multiple Gene Cassettes
Source: Front Bioeng Biotechnol. 2021 Dec 10;9:797521. doi: 10.3389/fbioe.2021.797521 (PMC8703077; doi:10.3389/fbioe.2021.797521)
Supplement: Supplementary file 1 [file DataSheet1.docx]

Supplementary Material

Introduction of modified BglBrick system in *Lactococcus lactis* for straightforward assembly of multiple gene cassettes

Tina Vida Plavec^1^, Tim Ključevšek^1,2^, Aleš Berlec^1,2*^

^1^ Department of Biotechnology, Jožef Stefan Institute, Jamova 39, Ljubljana, Slovenia

^2^ University of Ljubljana, Faculty of Pharmacy, Aškerčeva 7, Ljubljana, Slovenia


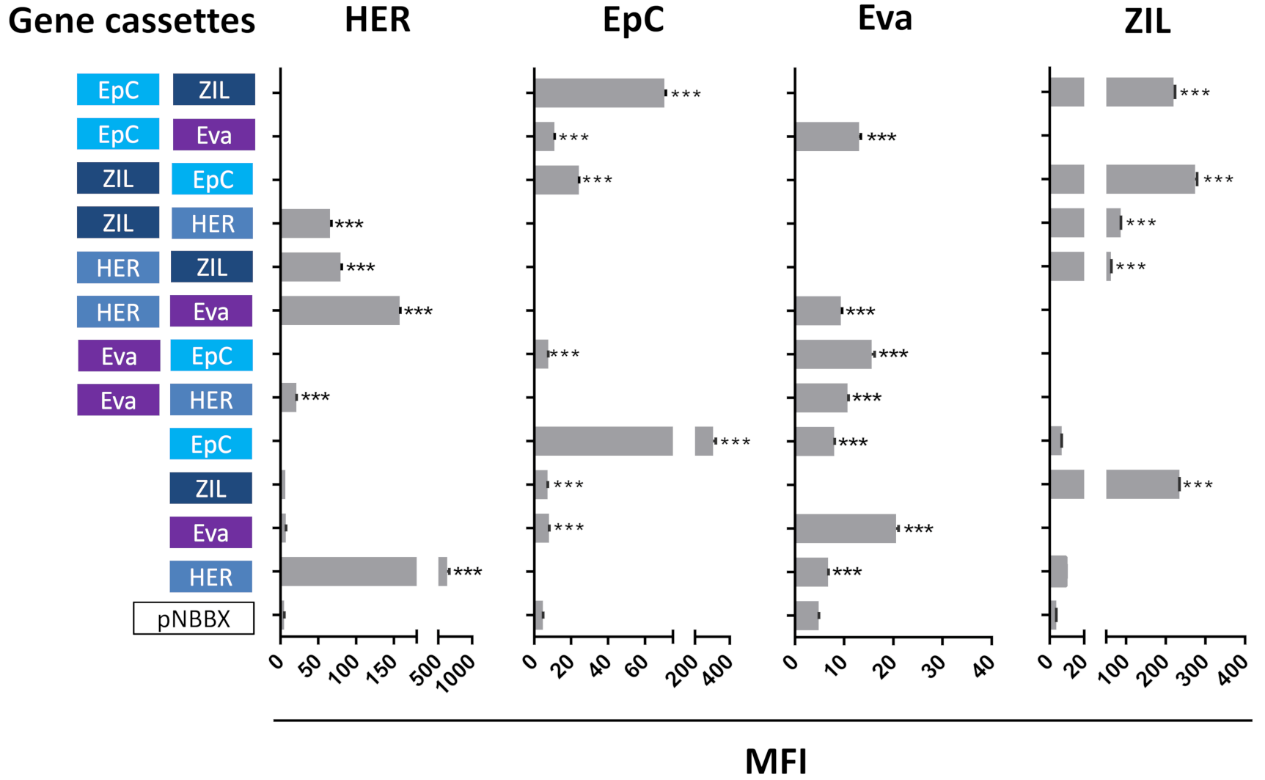


Figure S1: Surface display of individual protein binders (cassettes HER, EpC, Eva and ZIL) assessed by flow cytometry (mean fluorescence intensity, MFI in *L. lactis* containing pNBBX with with up to two gene cassettes (denoted in 5’-3’ direction). ***P < 0.0001.


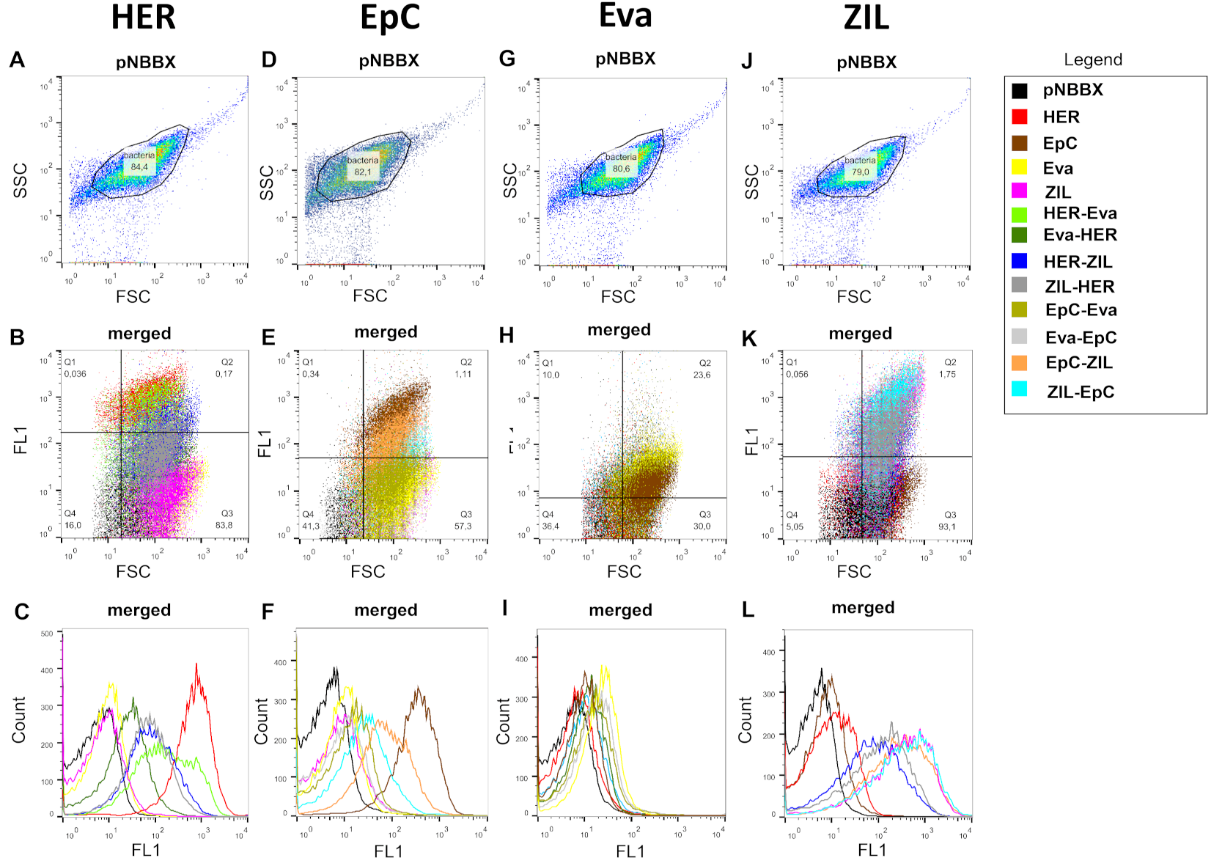


Figure S2. Expanded flow cytometry data that is summarized in Figure S1. (A, D, G, J) Gating strategy on the example of *L. lactis* containing the control plasmid pNBBX presented as dot plot display of forward scatter (FSC) versus side scatter (SSC). (B, E, H, K) Fluorescence of *L. lactis* cells expressing one or two gene cassettes presented as dot plot display of FSC versus FL1, or as histogram demonstrating characteristic shift (C, F, I, L). Data on individual binding proteins (HER, EpC, Eva, ZIL) is assembled in columns.


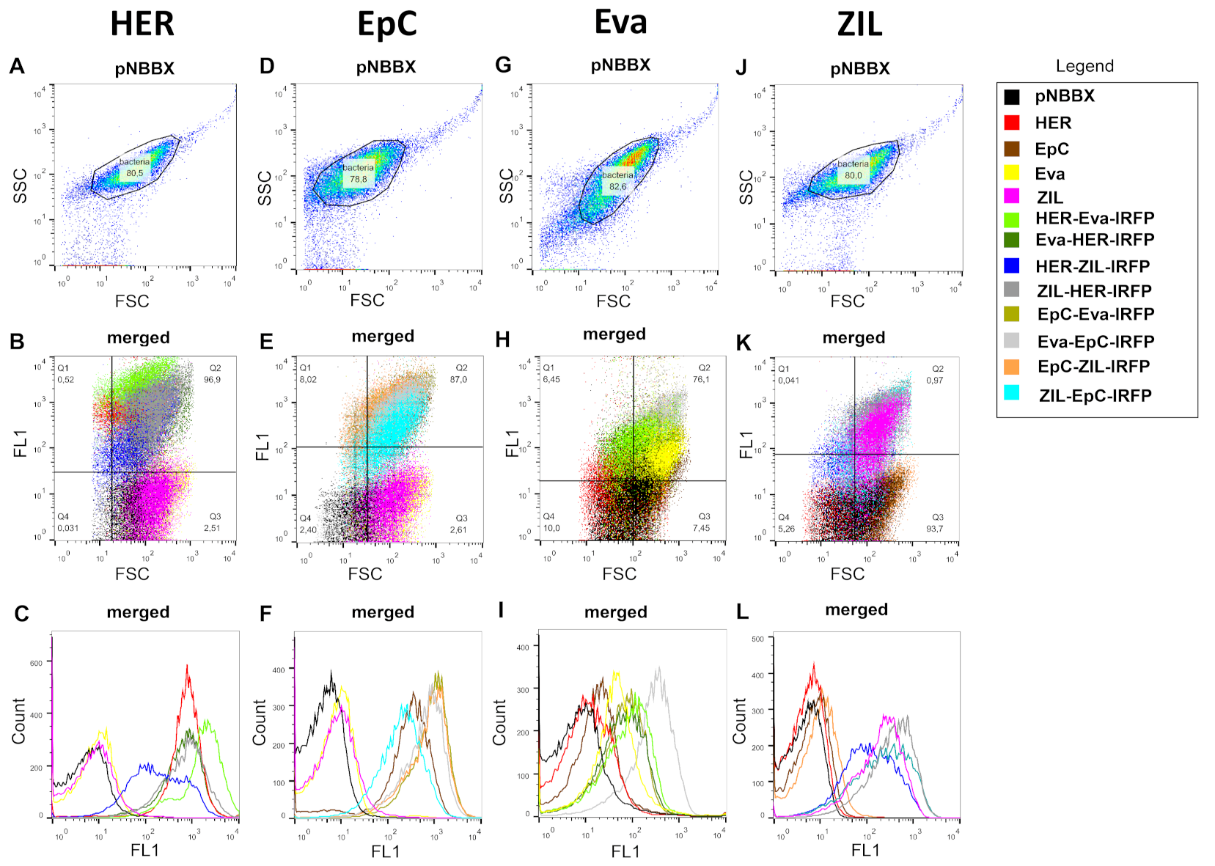


Figure S3. Expanded flow cytometry data that is summarized in Figure 3. (A, D, G, J) Gating strategy on the example of *L. lactis* containing the control plasmid pNBBX presented as dot plot display of forward scatter (FSC) versus side scatter (SSC). (B, E, H, K) Fluorescence of *L. lactis* cells expressing one or three gene cassettes presented as dot plot display of FSC versus FL1, or as histogram demonstrating characteristic shift (C, F, I, L). Data on individual binding proteins (HER, EpC, Eva, ZIL) is assembled in columns.


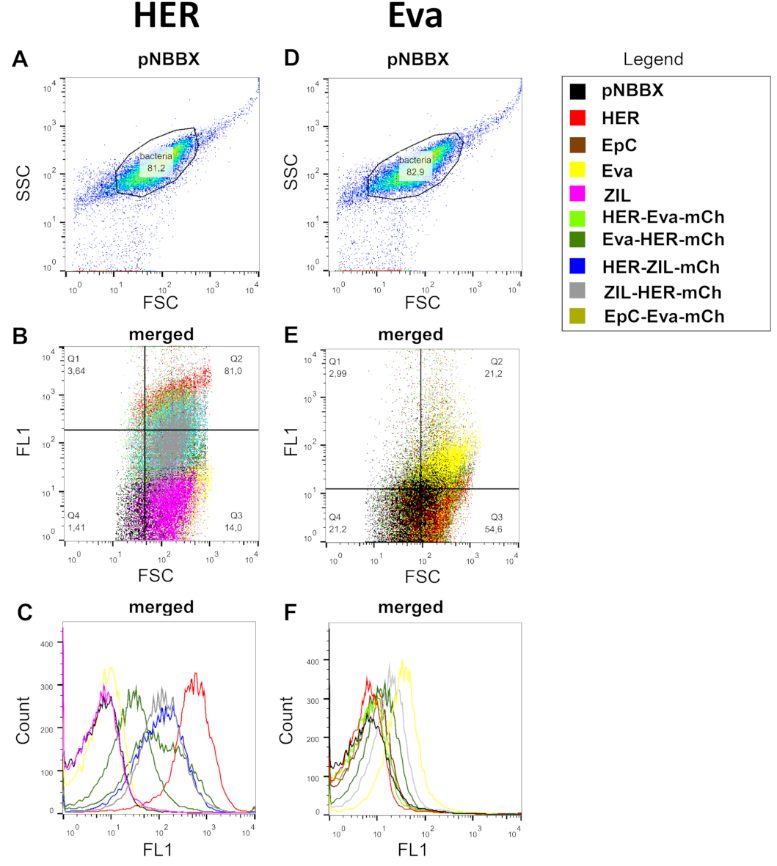


Figure S4. Expanded flow cytometry data that is summarized in Figure 4. (A, D) Gating strategy on the example of *L. lactis* containing the control plasmid pNBBX presented as dot plot display of forward scatter (FSC) versus side scatter (SSC). (B, E) Fluorescence of *L. lactis* cells expressing one or three gene cassettes presented as dot plot display of FSC versus FL1, or as histogram demonstrating characteristic shift (C, F). Data on individual binding proteins (HER, EpC) is assembled in columns.
